# Comparing the prevalence and influencing factors of postpartum depression in primiparous and multiparous women in China

**DOI:** 10.3389/fpsyt.2024.1479427

**Published:** 2024-10-04

**Authors:** Jing Zhang, Peipei Wang, Weisen Fan, Cuixia Lin

**Affiliations:** ^1^ School of Health, Shandong University of Traditional Chinese Medicine, Jinan, Shandong, China; ^2^ Medical department, Kashi Prefectural Hospital of Traditional Chinese Medicine, Kashi, Xinjiang, China; ^3^ Department of Traditional Chinese Medicine, Dongchangfu Maternal and Child Health Hospital, Liaocheng, Shandong, China; ^4^ Department of Gynaecology, China Academy of Chinese Medical Sciences, Beijing, China

**Keywords:** postpartum depression, primiparous, multiparous, prevalence, influencing factors, China

## Abstract

**Background:**

Few studies have compared the influencing factors of postpartum depression between primiparous and multiparous women. Therefore, this study is aimed to investigate the prevalence and influencing factors of postpartum depression in primiparous and multiparous women, and provide reference suggestions for clinical nursing.

**Methods:**

A total of 429 postpartum women who gave birth at a Maternal and Child Health Hospital in Shandong Province, China, from April to June 2024, were recruited by convenience sampling. A Sociodemographic Questionnaire, Edinburgh Postpartum Depression Scale, Pittsburgh Sleep Quality Index, and Perceived Social Support Scale were used for investigation. SPSS 26.0 was used to analyze the data, and multivariate regression was employed to analyze the influencing factors of postpartum depression between primiparous and multiparous women.

**Results:**

The total prevalence of postpartum depression among 429 postpartum women (191 primiparas and 238 multiparas) was 22.14%. The prevalence of postpartum depression among primiparous and multiparous women were 21.99% and 22.27%, respectively, with no statistically significant difference [OR=1.016, 95% CI (0.642, 1.608)]. Sleep quality is a common protective factor for postpartum depression in both primiparous and multiparous women, while perceived social support is another protective factor for multiparous women. The risk factors are different in both two group, there is no risk factor found in primiparous women, the newborns health and women’s expectation on newborns gender are risk factors for postpartum depression in multiparous women.

**Conclusions:**

Both primiparous and multiparous women have a high risk of postpartum depression. In order to promote the mental health of postpartum women, precise nursing measures should be adopted for different parity of postpartum women in clinical practice.

## 1 Introduction

Childbirth is a significant psychological trauma experienced by women throughout their lives. The intense pain, loss of dignity, forgotten or ignored by family members, and role changes during childbirth can all lead to postpartum depression (PPD) (1). The prevalence of postpartum depression is particularly high during pregnancy (2) and in the first 15 months postpartum (3), mainly manifested as depression, frustration, sadness, crying, irritability, excitement, poor coping ability, etc. (4). However, postpartum depression is becoming a public health problem, it not only seriously affects the physical and mental health, but also increases the hospitalization and mortality rates of offspring (5).

A recent meta-analysis review from University of California estimated that postpartum depression affects about 19.18% of women worldwide (6), higher than the prevalence rate of 17.22% in 2021 (7). Moreover, the prevalence rates of postpartum depression vary in countries and regions due to different culture, perception, economic environments, etc. Previous studies have shown that the prevalence rate was significantly lower in developed countries or high-income areas (8, 9) like the United States (11%) (10), Australia (12%) (11), and Europe (8%) (12), compared to the developing countries or low-income areas. And the highest prevalence rate was found in Southern Africa, which was 39.96% (7). The prevalence of postpartum depression in China is 21.4%, showing a rising trend (8, 13).

Postpartum depression is influenced by various factors, including sociodemographic factors, obstetric factors, infant characteristics, and social environmental factors (14, 15). Among them, the sociodemographic factors associated with significantly increasing the risk of postpartum depression are older or younger age, lower education level, lower family income (16–18). In addition, other factors such as pregnancy complications, premature birth, lower social support, poor marital relationship (19–21) were reported as the risk factors related to postpartum depression. Also, poor sleep quality (18, 20) can lead to postpartum depression. Even though most of the identified risk factors for postpartum depression are believed to be preventable by appropriate, timely action, however, failure to recognize the differences in the impact of these factors on primiparous and multiparous women result in ineffective intervention.

In fact, all postpartum women face significant physical and psychological challenges, previous studies have not reached a consistent conclusion on the prevalence of postpartum depression between primiparous and multiparous women, which may be related to cultural and behavioral differences. The first-child pregnancy means accepting a transition to motherhood for primiparous women, and the lack of parenting experience for new mothers can increase the difficulty of parent-child interaction (22). There are also negative changes in marital relationships (23) and body image (24) during first-child pregnancy, increasing the risk of postpartum depression for new mothers. Moreover, multiparous women may face new pressures, they need to balance the energy of caring for multiple children to be good mothers, reducing self-blame and ashamed (25). They may also experience serious pregnancy complications and increase risk of adverse pregnancy outcomes (26, 27), which can affect their quality of life and mental health (28).

With the relaxation of birth policy in 2021, the proportion of second-child and elderly mothers has increased, the prevalence of postpartum depression and influencing factors for primiparous and multiparous women remains unclear. Therefore, this study aims to explore the prevalence and influencing factors of postpartum depression in primiparous and multiparous women, provide nursing reference suggestions for clinical nursing staffs and enhance the awareness of postpartum women and their families to pay attention to mental health in prenatal and postpartum period.

## 2 Materials and methods

### 2.1 Participants

Using convenience sampling, 429 participants who gave birth at a Maternal and Child Health Hospital in Shandong Province, China, from April to June 2024, were selected. The inclusion criteria were: (1) women aged 18 and higher, (2) women who have a certain understanding ability and be able to independently complete questionnaire filling, (3) informed consent and voluntary participating in this study. The exclusion criteria were: (1) suffering from severe physical or mental disorders, (2) those who have incomplete questionnaire information.

### 2.2 Study design and data collection

This study used a cross-sectional study design to collect questionnaire information from postpartum women. The QR code of questionnaire, posted on Questionnaire Star Platform (https://www.wjx.cn/), was sent to postpartum women. The researcher explained the study purpose to the participants and guided them to independently complete the questionnaire within 30 minutes after obtaining the QR code, ensuring that the questionnaire is filled out completely. If there were any omissions or ambiguous answers, the participants should promptly supplement them.

### 2.3 Measurement

#### 2.3.1 Sociodemographic questionnaire

The self-designed questionnaire included age, place of residence, status of employment during pregnancy, education level, annual household income, marriage age, cigarette smoking, alcohol consumption, pregnancy intention, routine prenatal examination, history of abnormal pregnancy, gestational week, delivery mode, delivery time, newborns health, women’s expectations on newborns gender, husband’s expectations on newborns gender, feeding patterns.

#### 2.3.2 Edinburgh postpartum depression scale

The EPDS was developed by Cox et al. (29) in 1978 and localized by Lee et al. (30), it was used for the assessment of postpartum depression in pregnant women. There are a total of 10 items, including mood, pleasure, self-accusation, anxiety, fear, insomnia, coping ability, sadness, crying, and self-inflicted injury. Each item has a score of 4 levels (never=0 points, occasionally=1 point, frequently=2 points, always=3 points), of which 2 items are scored in reverse. The total scores range from 0 to 30 points, with higher scores indicating more severe depression. A score of 10 points and higher is considered as a cut-off for postpartum depression. In this study, the Cronbach’s alphas of the scale are 0.875, indicating good internal consistency.

#### 2.3.3 Pittsburgh sleep quality index

The PSQI was mainly used to evaluate the sleep status of participants in the past month (31). The PSQI is a self-report scale with 18 questions, rating from 7 dimensions including sleep latency, habitual sleep efficiency, sleep persistence, subjective sleep quality, sleep disorders, hypnotic drug use, and daytime dysfunction. Each dimension is scored on a scale of 0-3 points, with a score range of 0-21 points. The higher the score, the worse the sleep quality. When using the PSQI, a score of ≥8 indicates poor sleep quality. In this study, the Cronbach’s alphas of the scale are 0.715.

#### 2.3.4 Perceived social support scale

The PSSS was used to assess support from three dimensions (4 items each)- family support, friend support, and significant other support (32). The scale contains 12 items made on a 7-point Likert-type scale ranging from 1 (very strongly disagree) to 7 (very strongly agree). Raw scores are converted from 12 to 84 points. The higher the score, the higher the perceived level of social support. 12-36 points mean low support, 37-60 points mean middle support, 61-84 points mean high support. In this study, the Cronbach’s alphas of the scale are 0.966, indicating good internal consistency.

### 2.4 Statistical analysis

IBM SPSS Statistics version 26.0 was used for data analysis. Numerical measurements were described as the mean and standard deviation (SD), while categorical measurements were presented as numbers and percentages (%). Univariate analyses, including t-tests and chi-square tests, were employed to assess potential relationships between the demographic variables, sleep quality, perceived social support of participants and postpartum depression. Multivariate logistic regression analysis was used to identify the influencing factors of postpartum depression. The threshold for determining statistical significance was set at P<0.05.

### 2.5 Ethics approval

This study was conducted in strict adherence to the guidelines set out in the Helsinki Declaration and approved by the Ethics Review Committee of Dongchangfu Maternal and Child Health Hospital, Liaocheng, China (approval number 20240523). Informed consent was obtained from all participants before filling in the questionnaire.

## 3 Results

### 3.1 Comparison of sociodemographic characteristics in postpartum women

A total of 436 questionnaires were distributed, and 429 valid questionnaires were collected in this study, with an effective response rate of 98.39%. Of the 429 participants involved in this study, they were aged 18 to 55 years old, with an average of (28.91 ± 5.23) years old, and their gestational weeks were from 28 to 44.57 weeks, with an average of (39.00 ± 1.46) weeks.

There were 191 primiparous women and 238 multiparous women in this study. For 191 primiparous women, their average ages were (25.98 ± 4.41) years old, the average gestational week is (39.27 ± 1.53) weeks, 101(52.88%) lived in urban area, 102(53.40%) had no work during pregnancy, 75(39.27%) were junior college, 96(50.26%) had an annual household income of 50000-100000 yuan, and 113(59.16%) got married for 1-3 years. Among them, 186(97.38%) had no cigarette smoking, 187(97.91%) had no alcohol consumption, 126(65.97%) were planned pregnancy, 159(83.25%) had routine prenatal examination, 24(12.57%) had a history of abnormal pregnancy, 109(57.07%) were vaginal delivery and 78(40.84%) were cesarean section, 103(53.93%) had the delivery time of ≤1 hour, 186(97.38%) had a healthy baby, 184(96.34%) women and 185(96.86%) husbands were satisfied with the gender of the baby, 106(55.50%) were mixed feeding and 77(40.31%) breastfeeding. Of the 238 multiparous women, their average ages were (31.26 ± 4.63) years old, the average gestational week is (38.78 ± 1.37) weeks, 72(30.25%) were bachelor’ s degree and above, 206(86.55%) got married for more than 3 years, 218(91.60%) had routine prenatal examination, 63(26.47%) had a history of abnormal pregnancy, 133(55.88%) were cesarean section and 102(42.86%) were vaginal delivery, 125(52.52%) were breastfeeding and 100(42.02%) mixed feeding. Others had the similar percentage with primiparous women. Therefore, there were statistically significant differences between primiparous and multiparous women in terms of age, gestational week, education level, marriage age, routine prenatal examination, history of abnormal pregnancy, delivery mode, and feeding patterns (all P<0.05), while there were no statistically significant differences in other variables (all P>0.05), as shown in **Table 1**.

**Table 1:** Comparison of sociodemographic characteristics in postpartum women (N=429).

Variables, M ± SD or n (%)	Primiparas (n=191)	Multiparas (n=238)	t/χ^2^	p-Value
Age	25.98 ± 4.41	31.26 ± 4.63	-12.057	0.000*
Gestational week	39.27 ± 1.53	38.78 ± 1.37	3.521	0.000*
Place of residence			0.074	0.785
Rural area	90 (47.12)	109 (45.80)		
Urban area	101 (52.88)	129 (54.20)		
Status of employment during pregnancy			0.491	0.483
Yes	89 (46.60)	119 (50.00)		
No	102 (53.40)	119 (50.00)		
Education level			26.193	0.000*
Junior high school and below	14 (7.33)	54 (22.69)		
High school/secondary school	29 (15.18)	51 (21.43)		
Junior college	75 (39.27)	61 (25.63)		
Bachelor’ s degree and above	73 (38.22)	72 (30.25)		
Annual household income			3.188	0.364
Below 50000 yuan	55 (28.80)	72 (30.25)		
50000~100000 yuan	96 (50.26)	109 (45.80)		
100000~200000 yuan	34 (17.80)	41 (17.23)		
Over 200000 yuan	6 (3.14)	16 (6.72)		
Marriage age			267.368	0.000*
Less than 1 year	63 (32.98)	3 (1.26)		
1~3 years	113 (59.16)	29 (12.18)		
More than 3 years	15 (7.85)	206 (86.55)		
Cigarette smoking			2.086	0.149
Yes	5 (2.62)	2 (0.84)		
No	186 (97.38)	236 (99.16)		
Alcohol consumption			0.085	0.771
Yes	4 (2.09)	6 (2.52)		
No	187 (97.91)	232 (97.48)		
Pregnancy intention			0.008	0.928
Yes	126 (65.97)	158 (66.39)		
No	65 (34.03)	80 (33.61)		
Routine prenatal examination			6.937	0.008*
Yes	159 (83.25)	218 (91.60)		
No	32 (16.75)	20 (8.40)		
History of abnormal pregnancy			12.673	0.000*
Yes	24 (12.57)	63 (26.47)		
No	167 (87.43)	175 (73.53)		
Delivery mode			9.679	0.008*
Vaginal delivery	109 (57.07)	102 (42.86)		
Assisted vaginal delivery	4 (2.09)	3 (1.26)		
Cesarean section	78 (40.84)	133 (55.88)		
Delivery time			3.165	0.205
≤1 hour	103 (53.93)	108 (45.38)		
1~3 hours	74 (38.74)	111 (46.64)		
≥3 hours	14 (7.33)	19 (7.98)		
Newborns health			0.041	0.840
Yes	186 (97.38)	231 (97.06)		
No	5 (2.62)	7 (2.94)		
Women’s expectations on newborns gender			0.770	0.380
Yes	184 (96.34)	225 (94.54)		
No	7 (3.66)	13 (5.46)		
Husband’s expectations on newborns gender			0.459	0.498
Yes	185 (96.86)	233 (97.90)		
No	6 (3.14)	5 (2.10)		
Feeding patterns			7.715	0.021*
Breastfeeding	77 (40.31)	125 (52.52)		
Formula feeding	8 (4.19)	13 (5.46)		
Mixed feeding	106 (55.50)	100 (42.02)		

*p<0.05 for all tables.

### 3.2 Prevalence of postpartum depression in postpartum women

Among the 429 postpartum women included, a total of 95 experienced postpartum depression, with a total prevalence of 22.14%. The prevalence of postpartum depression in primiparous and multiparous women were 21.99% and 22.27%, respectively. After adjusting for variables with statistically significant differences between the two groups of postpartum women, the results showed that there was no statistically significant difference in the prevalence of postpartum depression between primiparous and multiparous women [OR=1.016, 95% CI (0.642, 1.608)] (**Table 2**).

**Table 2:** Prevalence of postpartum depression in postpartum women (N=429).

Variables, n (%)	Primiparas(n=191)	Multiparas(n=238)	p-Value
EPDS≥10	42 (21.99)	53 (22.27)	0.945
EPDS<10	149 (78.01)	185 (77.73)	

### 3.3 PSQI and PSSS scores in postpartum women

PSQI total score of primiparous and multiparous women were (5.66 ± 2.95) points and (5.62 ± 3.48) points, respectively, with no statistically significant differences (P>0.05). The sleep latency scores of primiparous and multiparous women in PSQI were (1.29 ± 0.91) points and (1.07 ± 0.89) points, respectively, with statistically significant differences (t=2.524, P<0.05); there were no statistically significant differences in other six factors (all P>0.05). Based on the PSQI score of ≥ 8, this study classified sleep quality into poor (PSQI ≥ 8) and good (PSQI < 8). Among them, 141(73.82%) of primiparas and 172(72.27%) of multiparas had a good sleep quality, with no statistically significant differences (P>0.05) (**Table 3**).

**Table 3:** Scores of Pittsburgh Sleep Quality Index (PSQI) in postpartum women (N=429).

Variables, M ± SD or n (%)	Primiparas(n=191)	Multiparas(n=238)	p-Value
Subjective sleep quality	1.15 ± 0.68	1.14 ± 0.80	0.900
Sleep latency	1.29 ± 0.91	1.07 ± 0.89	0.012*
Sleep persistence	0.23 ± 0.60	0.30 ± 0.69	0.218
Habitual sleep efficiency	0.48 ± 0.78	0.64 ± 0.97	0.056
Sleep disorders	1.37 ± 0.66	1.29 ± 0.68	0.217
Hypnotic drug use	0.02 ± 0.16	0.00 ± 0.00	0.180
Daytime dysfunction	1.14 ± 0.98	1.18 ± 1.03	0.649
PSQI total score	5.66 ± 2.95	5.62 ± 3.48	0.892
Poor sleep quality	50(26.18)	66 (27.73)	0.719
Good sleep quality	141(73.82)	172 (72.27)	

*p<0.05 for all tables.

PSSS total score of primiparous and multiparous women were (68.32 ± 13.26) points and (63.47 ± 14.51) points, respectively, with statistically significant differences (P<0.05). The scores of family support were (23.46 ± 4.67) points in primiparas and (22.05 ± 5.24) points in multiparas, friend support were (22.58 ± 4.84) points in primiparas and (20.71 ± 5.45) points in multiparas, other support were (22.29 ± 4.70) points in primiparas and (20.71 ± 4.92) points in multiparas. There were statistically significant differences (P<0.05) in the scores of family support, friend support, and other support between primiparous and multiparous women. According to the scoring criteria, perceived social support was divided into low support, middle support and high support. 136(71.20%) of primiparas and 135(56.72%) of multiparas had high support, and there were statistically significant differences between primiparous and multiparous women (P<0.05) (**Table 4**).

**Table 4:** Scores of Perceived Social Support Scale (PSSS) in postpartum women (N=429).

Variables, M ± SD or n (%)	Primiparas(n=191)	Multiparas(n=238)	p-Value
Family support	23.46 ± 4.67	22.05 ± 5.24	0.004*
Friend support	22.58 ± 4.84	20.71 ± 5.45	0.000*
Other support	22.29 ± 4.70	20.71 ± 4.92	0.001*
PSSS total score	68.32 ± 13.26	63.47 ± 14.51	0.000*
Low support	2 (1.05)	8 (3.36)	0.005*
Middle support	53 (27.75)	95 (39.92)	
High support	136 (71.20)	135 (56.72)	

*p<0.05 for all tables.

### 3.4 Univariate analysis and multivariate logistic regression analysis of postpartum depression

As shown in **Table 5**, univariate analysis was carried out to determine the association of independent variables with postpartum depression. EPDS≥10 was called postpartum depression, EPDS<10 was healthy. For primiparous women, 20(47.62%) were postpartum depression with poor sleep quality and 30(20.13%) were healthy with poor sleep quality. 23(54.76%) and 18(42.86%) were high support and middle support in primiparous women with postpartum depression, while 113(75.84%) and 35(23.49%) were in healthy women. Therefore, the significant variables were sleep quality and perceived social support in 191 primiparous women (all P<0.05). Similarly, there were statistically significant differences in age, cigarette smoking, alcohol consumption, planned pregnancy, newborns health, women’s expectations on newborns gender, sleep quality, and perceived social support in 238 multiparous women (all P<0.05).

**Table 5:** Univariate analysis of postpartum depression in postpartum women (N=429).

Variables, M ± SD or n (%)	EPDS≥10	EPDS<10	t/χ^2^	p-Value
Primiparas (n=191)				
Sleep quality			12.808	0.000*
Poor	20 (47.62)	30 (20.13)		
Good	22 (52.38)	119 (79.87)		
Perceived social support			7.388	0.025*
Low support	1 (2.38)	1 (0.67)		
Middle support	18 (42.86)	35 (23.49)		
High support	23 (54.76)	113 (75.84)		
Multiparas (n=238)				
Age	29.70 ± 5.43	31.71 ± 4.28	-2.491	0.015*
Cigarette smoking			7.040	0.008*
Yes	2 (3.77)	0 (0.00)		
No	51 (96.23)	185 (100.00)		
Alcohol consumption			13.259	0.000*
Yes	5 (9.43)	1 (0.54)		
No	48 (90.57)	184 (99.46)		
Pregnancy intention			4.161	0.041*
Yes	29 (54.72)	129 (69.73)		
No	24 (45.28)	56 (30.27)		
Newborns health			10.069	0.002*
Yes	48 (90.57)	183 (98.92)		
No	5 (9.43)	2 (1.08)		
Women’s expectations on newborns gender			4.532	0.033*
Yes	47 (88.68)	178 (96.22)		
No	6 (11.32)	7 (3.78)		
Sleep quality			45.127	0.000*
Poor	34 (64.15)	32 (17.30)		
Good	19 (35.85)	153 (82.70)		
Perceived social support			19.568	0.000*
Low support	3 (5.66)	5 (2.70)		
Middle support	34 (64.15)	61 (32.97)		
High support	16 (30.19)	119 (64.32)		

*p<0.05 for all tables.

Taking the significant variables in univariate analysis as independent variables and postpartum depression as the dependent variable, a multivariate logistic regression analysis was used to identify the relative factors affected postpartum depression. The results showed that there were statistically significant differences in sleep quality in primiparous women [OR=0.300, 95% CI (0.142, 0.634)] (P<0.05). The newborns health [OR=8.886, 95% CI (1.204, 65.607)], women’s expectations on newborns gender [OR=4.959, 95% CI (1.170, 21.019)], sleep quality [OR=0.130, 95% CI (0.059, 0.285)] and perceived social support [OR=0.375, 95% CI (0.170, 0.827)] are the factors with statistically significant differences for postpartum depression in multiparous women (all P<0.05) (**Table 6**). **Tables 5**, **6** only lists the factors statistically significant.

**Table 6:** Multivariate logistic regression analysis of postpartum depression (N=429).

Variables	b	Sb	Wald χ^2^	p-Value	OR	95%CI
Primiparas (n=191)						
Constants	1.931	0.270	51.259	0.000*	6.895	
Sleep quality	-1.203	0.381	9.957	0.002*	0.300	(0.142, 0.634)
Multiparas (n=238)						
Constants	-3.420	1.886	3.288	0.070	0.033	
Newborns health	2.185	1.020	4.587	0.032*	8.886	(1.204, 65.607)
Women’s expectations on newborns gender	1.601	0.737	4.723	0.030*	4.959	(1.170, 21.019)
Sleep quality	-2.042	0.402	25.807	0.000*	0.130	(0.059, 0.285)
Perceived social support						
Perceived social support (1)	-1.579	1.002	2.482	0.115	0.206	(0.029, 1.470)
Perceived social support (2)	-0.981	0.403	5.914	0.015*	0.375	(0.170, 0.827)

(1) Low support compared to high support; (2) Middle support compared to high support.

*p<0.05 for all tables.

## 4 Discussion

In this study, the total prevalence of postpartum depression was 22.14%, this is nearly consistent with the results of Ng et al. (33), which found a prevalence of 22%. However, it was higher than previous studies conducted in United States (11%) (10) and Europe (8%) (12), this may be related to cultural environment, different screening scales and cutoff values. According to the previous study from France, the prevalence of postpartum depression was 9.9% with a cutoff value of 13, but 15.5% with a cutoff value of 11 (34). A score of 10 points and higher is considered as a cut-off value in this study, previous studies found that the 9/10 cut-off point is substantially better than the conventional 12/13 cut-off point in detecting depression in China, otherwise, it will yield an unsatisfactorily high rate of false negatives (30). Therefore, 12/13 cut-off point is usually used abroad, 9/10 cut-off point is for China.

The prevalence of postpartum depression in primiparous and multiparous women were 21.99% and 22.27%, respectively, both at a relatively high level. This may be because primiparous women are not yet able to adapt well to their new identity, lack parenting experience, and cause conflicts between mother-in-law and daughter-in-law, which leads to excessive psychological pressure and depression; multiparous women have some parenting experience, but it is difficult for them to balance the time and energy between two or more children, which also leads to fatigue and guilt. In addition, being older can increase the risk of postpartum depression (35). There was no statistically significant difference in the prevalence of postpartum depression between primiparous and multiparous women in this study, which cannot clearly indicate that primiparous or multiparous women have a higher risk of postpartum depression. This is consistent with the findings of Huang et al. (36). Therefore, if a mother is concerned that having a second or third child will increase the risk of postpartum depression, obstetricians can truthfully inform the mother of this research conclusion, which can help the mother relax and relieve the pressure of childbirth.

This study found that the health of newborns is a risk factor for postpartum depression in multiparous women, which is consistent with the findings of Dragomir et al. (37) and Chen et al. (38). Compared with those of healthy newborns, the women whose newborns are sick or admitted to the neonatal intensive care unit (NICU) will bear more psychological pressure, including adapting to sick newborns, physical and emotional separation from their children, fear and frustration of childbirth, etc (39). For multiparous women, it is more difficult to take good care of a sick baby and other child. Therefore, they may experience more depression. Moreover, this study also showed a statistically significant difference in history of abnormal pregnancy between primiparous and multiparous women, indicating that multiparous women may have heightened concerns about the health of their newborns due to past abnormal pregnancy experiences. Therefore, obstetricians need to popularize the necessity of routine prenatal examination to women, timely track the health status of newborns, and provide comprehensive knowledge of health care on newborns to reduce the risk of postpartum depression caused by newborns health.

It was found that whether the gender of the newborns meets women’s expectations is a risk factor for postpartum depression in multiparous women. Previous studies have shown that women who give birth to boys have higher levels of depression (40). Some multiparous women may be deeply influenced by the traditional Chinese concept of “favoring sons over daughters” and have higher expectations for the gender of the newborns. Unexpected newborns gender may exacerbate the emotional and economic pressure of multiparous women, and some may face criticism (41), which may even mean a new pregnancy (42). Primiparous women are not deeply affected by their expectations on newborns gender, possibly because they can still bear the economic pressure of raising their first child and do not have a strong aversion to new pregnancies. Therefore, obstetricians should carry out ideological education on gender equality and help women establish a good mentality, reducing the negative influence caused by gender expectations. In addition, earlier newborns gender identification (43) may help them prepare well psychologically in advance and reduce the risk of postpartum depression in multiparous women.

Sleep quality is a common protective factor for postpartum depression in primiparous and multiparous women. The lower the sleep quality, the higher the risk of postpartum depression, which is consistent with previous research (44, 45). Due to postpartum infant care, nighttime feeding and other reasons, women may experience varying degrees of sleep problems in the early postpartum period. Lack of sleep can lead to emotional disorders and increase the risk of postpartum depression. Studies have shown that postpartum depression and sleep quality may interact with each other, with women experiencing postpartum depression having poorer sleep quality (46). Both primiparous and multiparous women should recognize the importance of sleep quality, coordinate the schedules of sleep and feeding with their partners. Obstetricians and nursing staffs should provide effective strategies to improve sleep quality for postpartum women. In addition, women and their families should learn some techniques to relieve emotions and avoid postpartum depression.

This study also found that perceived social support is a protective factor for postpartum depression in multiparous women. The more social support perceived, the lower the risk of postpartum depression, which is consistent with previous studies (41, 47). Studies have shown that if social support is insufficient, the risk of postpartum depression for multiparous women is five times higher (48). For multiparous women, giving birth again requires greater physical challenges and psychological pressure, as well as the responsibility of taking care of multiple children. They cannot receive the same level of social support as their first childbirth, therefore they need more psychological support from their families, friends and society, especially from their partners (49). If the marital relationship is poor or their partners are not mature enough to take responsibility, postpartum women cannot receive good emotional support, which can easily lead to feelings of helplessness and despair, and even postpartum depression. In this study, primiparous women received more social support, which may be because their family members giving more attention and support to women and child for the joy and caution of their first childbirth. Therefore, nursing staffs should strengthen the family members’ (especially their partners) awareness of providing psychological support and guide them to share parenting pressure together, providing a good family atmosphere for postpartum women and reducing the risk of postpartum depression.

In addition, previous studies found the relationship between postpartum depression and feeding patterns (50), the postpartum women who maintain breastfeeding have a lower risk for postpartum depression than those who feed formula to their infants. Although there are significant differences in feeding patterns between primiparous and multiparous women in this study, we did not find any differences between postpartum depression and feeding patterns. Some studies also found that delivery mode (51) is associated with the occurrence of postpartum depression. Prevalence of postpartum depression was significantly lower in the normal vaginal delivery group than those of the emergency and elective caesarian section groups. However, no any significant differences were found in this study. A meta-analysis showed the effect of perinatal smoking or drinking (52) on postpartum depression. However, perinatal smoking and drinking were not independent influencing factors for postpartum depression in this study. Moreover, even though not specifically evaluated in this study, previous studies reported the significant association between postpartum depression and a family psychiatric history (53). The daughters had the higher depressive symptom scores than their mothers’ generation (54). In the same way, there are some factors that we did not find to be associated with postpartum depression in this study, such as pregnancy intention, routine prenatal examination, gestational week, delivery time and the history of abnormal pregnancy, which does not mean that they cannot affect the occurrence of postpartum depression. This may be due to the influence of traditional culture, where many participants have a certain sense of shame towards mental illness, leading to false reporting when filling out questionnaires. It may also be related to the sample size or cutoff scores of the EPDS.

This study provides a new approach for early depression screening in women during the perinatal period. By distinguishing the factors that affect postpartum depression in primiparous and multiparous women, targeted screening and psychological care can be provided for them. At the same time, the results of this study can verify the timeliness and population adaptability of commonly used screening scales, and develop a postpartum depression scale that is in line with the characteristics of contemporary Chinese women. There are some limitations in this study. The participants were only recruited from a certain hospital, which limits its representativeness and generalizability. Moreover, the questionnaire collection is only conducted within one week after delivery and cannot fully reflect the prevalence of diseases one year after delivery. The prevalence showed in this study may be lower than the actual situation. And the sample size is not large enough that there were no differences in the prevalence of postpartum depression and some variables do not have statistical significance between primiparous and multiparous women. In the future, the scope and sample size of the research can be expanded to obtain more accurate results.

## 5 Conclusion

The prevalence of postpartum depression in primiparous and multiparous women is at a relatively high level, and postpartum depression is influenced by factors such as sleep quality, perceived social support, newborns health, and women’s expectations on newborns gender. The mental health of postpartum women should be attracted the attention of the whole society. The government should work together with corresponding institutions to increase publicity and intervention on the mental health of postpartum women and extend paid maternity leave for women (35). Medical staffs should assist postpartum women and their families in learning more postpartum physical and mental care skills. The hospital should strengthen departmental cooperation to timely screen and provide psychological counselling for high-risk postpartum women, which may reduce the negative impact of postpartum depression on postpartum women and their families.

## Data Availability

The raw data supporting the conclusions of this article will be made available by the authors, without undue reservation.
